# Causality and the Interpretation of Epidemiologic Evidence

**DOI:** 10.1289/ehp.8297

**Published:** 2006-03-27

**Authors:** Michael Kundi

**Affiliations:** Institute of Environmental Health, Center for Public Health, Medical University of Vienna, Austria

**Keywords:** causality, epidemiology

## Abstract

There is an ongoing debate regarding how and when an agent’s or determinant’s impact can be interpreted as causation with respect to some target disease. The so-called criteria of causation, originating from the seminal work of Sir Austin Bradford Hill and Mervyn Susser, are often schematically applied disregarding the fact that they were meant neither as criteria nor as a checklist for attributing to a hazard the potential of disease causation. Furthermore, there is a tendency to misinterpret the lack of evidence for causation as evidence for lack of a causal relation. There are no criteria in the strict sense for the assessment of evidence concerning an agent’s or determinant’s propensity to cause a disease, nor are there criteria to dismiss the notion of causation. Rather, there is a discursive process of conjecture and refutation. In this commentary, I propose a dialogue approach for the assessment of an agent or determinant. Starting from epidemiologic evidence, four issues need to be addressed: temporal relation, association, environmental equivalence, and population equivalence. If there are no valid counterarguments, a factor is attributed the potential of disease causation. More often than not, there will be insufficient evidence from epidemiologic studies. In these cases, other evidence can be used instead that increases or decreases confidence in a factor being causally related to a disease. Even though every verdict of causation is provisional, action must not be postponed until better evidence is available if our present knowledge appears to demand immediate measures for health protection.

The principle of causality, so deeply embedded in humans’ minds that it has been thought of as immediately evident, is the very foundation not only of all three monotheistic world religions but also of the first staggering steps of science [*de nihilo nihil* (nothing can be born of nothing); Lucretius 1951]. Hume (1739) was the first to note that there is no logical foundation in the assumption that if in the past every event has had a cause, this will also be the case in the future and, furthermore, that what we perceive in daily life as well as in science is only a sequence of events but not cause and effect. Although Hume deeply believed in the truth of the principle of causality, he pointed to the role of the human mind in constructing reality and the futility of scientifically proving its validity. Kant (1791), as he became acquainted with Hume’s thoughts, was awakened from his metaphysical slumber, or so he kept saying, and set out to solve the problem of how Newton’s physics, which he thought of as eternally true, could be possible in the face of Hume’s demonstration that it cannot be inferred from experience. The Copernican turn in Kant’s reasoning was to imply the principle of causality from the assumption that it is among the conditions of every experience. Indeed, if A is a necessary condition of B, then B is a sufficient condition of A. Hence, if for every experience we make (B) it is a precondition that everything has a cause (A), then from the fact that we do have experiences (B), it follows that everything has a cause (A). However, to make this a logically coherent theory, Kant had to sacrifice “objective knowledge”—that is, the *Ding an sich* (the “thing in itself”) remains incomprehensible for the human mind. For more than 100 years, the philosophy of science circled around either the assumptions or the (untoward) consequences of Kant’s solution. When in 1905 Einstein published his special theory of relativity and his theory of the interaction of electrons and light (Einstein 1905a, 1905b), the very foundation of Kant’s philosophy was called into question: the universal truth of Newton’s mechanics (Newton 1726) and the validity of the deterministic concept. These considerations not only profoundly changed modern science but also resulted in an open-ended controversy within epistemology. And last but not least, epidemiology and the interpretation of epidemiologic evidence are deeply connected to these fundamental considerations about the nature of human knowledge.

## Defining Cause and Causality

The most advanced sciences, physics and chemistry, have altogether abandoned the concepts of cause and effect. These terms are no longer used in these sciences. Newton had already replaced cause and effect with functional relationships; however, to make himself understood to his contemporaries, in the third book of his *Principia* (1726) he spoke about causes (especially to defend his position of what can be called a minimal sufficient cause). Nevertheless, “cause and effect” remained terms used in physics, somewhat anachronistically, especially for scholarly purposes until the end of the 19th century. Mach (1883), alluding to Hume, stressed the psychological nature of these concepts and pointed out that “in nature there is no cause and no effect” and that these concepts are results of an economical processing of perceptions by the human mind.

The notion that diseases have natural causes and are not God’s punishments or trials or curses of malicious beings or results of supernatural forces has not even fully penetrated Western culture, let alone become the prevailing view worldwide. Despite its metaphysical character, the etiologic axiom that every disease has an endogenous and/or exogenous cause was extremely successful and is still the foundation of scientific medicine. However, what actually “causes” a disease has from the very beginning been a matter of controversy. Indeed, a single clinical phenomenon can have quite different “causes,” and one “cause” can have quite different clinical consequences (Table 1). These facts are not consistent with the original concept of causation, which states that a cause is an object that is followed by another, and where all objects similar to the first are followed by objects similar to the second (Hume 1739). Not even for infectious diseases does this (strong) concept of causation hold. (Hume gave several “definitions” of a cause, among these also what has been called the counter-factual approach, discussed below.)

How, then, should cause and causation be defined? In a review of definitions of “causation” in epidemiologic literature, Parascandola and Weed (2001) delineated five categories. However, all of these definitions (summarized in Table 1) have severe deficits. Not totally unexpected, the definitions found in the literature are insufficient to provide a basis for the notion of disease causation. As pointed out above for physical phenomena, it is also impossible for disease processes to draw an ontologic demarcation within the indefinite stream of events between causal and noncausal associations.

Consider a human being as a complex input–output system that is described by a path through a state space (of likely very high dimensionality) that may or may not explicitly depend on time. The task is to solve the equations that relate the input stream, the output stream, and the internal states to each other. The solution could give the probability that the human being will be in some internal state of disease at some point in time given a set of initial and/or side conditions. If we were in possession of such a tool, we would not need the crutch of a concept of causation. Meanwhile, in a pragmatic sense, it is reasonable to stay with this concept but hold in mind that it is just an economical way to organize the otherwise unfathomable stream of events and to take the necessary steps to counteract or prevent the disease process. The process of diagnosis itself is one of abstraction and generalization because no two diseased human beings given the same diagnosis have exactly the same features.

In this pragmatic sense, disease cause can be defined as follows: Given two or more populations of subjects that are sufficiently similar for the problem under study, a disease cause is a set of mutually exclusive conditions by which these populations differ that increase the probability of the disease. In some cases, the similarity must be high, such that only homozygous twins can be studied; in other cases, maybe only sex and age must be considered, or the state of immunity. To avoid encumbering the definition with unnecessary complexity, we use the term “conditions” and the active verb “increase.” What is meant is that a number of extrinsic and/or intrinsic factors (i.e., conditions) can be discerned that are present before diagnosis of the disease and that prevail at a time and for a duration that is compatible with what is known about the natural history of the disease. Hence, this temporal relation is a precondition for an agent to be considered a causal factor. The “conditions” must be mutually exclusive (e.g., groups of males characterized by one of the following conditions: smoking or having smoked cigarettes, cigars, pipes only, more than one of these, or none), because otherwise the increase in the probability of the disease cannot be uniquely related to any one of them.

This definition is in line with the main designs of epidemiologic studies: the cohort, the case–control, and the randomized controlled trial. It is also in line with the pragmatic definition that assessment of causality affords more than just the observation of an increased incidence or prevalence in some group or the other. This is the point from which Sir Austin Bradford Hill started his considerations that led to what are now commonly called the “Bradford Hill criteria” (1965).

## Taking Refuge in Causality

It seems that the first time causality entered the discussion on epidemiologic results was during the tobacco controversy in the late 1950s and early 1960s. In particular, the criticism of Fisher (1959) concerning the conclusions drawn from the British Doctors Study by Doll and Bradford Hill (1954) initiated a detailed consideration of the concept of causality that led to the famous presidential address by Bradford Hill to the Section of Occupational Medicine of the Royal Society of Medicine in 1965. In this talk, Bradford Hill discussed nine issues that should be addressed when deciding whether an observed association is a causal relationship. These issues, now called the “Bradford Hill criteria”—although they were not intended as criteria and not all of them have stood the test of time—are still the starting point of many a treatise on the subject today.

The Bradford Hill criteria were established such that, in the case they are met for a specific factor, this would increase our confidence in this factor being causally related to the disease. However, they were not intended to dismiss a factor as potentially causing the disease: “None of my nine viewpoints can bring indisputable evidence for or against the cause-and-effect hypothesis and none can be required as a *sine qua non*” (Bradford Hill 1965).

Some statements in the past few years about the relationship between environmental or occupational factors and human health have used the terms “causality” or “causal” in a negative sense—that is, claiming that there is no evidence for a causal relationship. First, one has to discriminate between evidence for no causal relationship, and no evidence of a causal relationship (Altman and Bland 1995). The former expresses an important piece of evidence that may have substantial consequences on steps taken to prevent health hazards, whereas the latter simply expresses lack of knowledge. It is, however, often misunderstood as an exculpation of the agent in question and is readily misused by interested parties to claim that exposure is not associated with adverse health effects.

Some examples of such statements illustrate the point:

- A “formal causation analysis based on an application of the Hill criteria confirms that there is no causal relationship between diesel exhaust and multiple myeloma” (Wong 2003).
- “Applying a weight-of-evidence evaluation to the PCB [polychlorinated biphenyl] epidemiologic studies can only lead to the conclusion that there is no causal relationship between PCB exposure and any form of cancer” (Golden et al. 2003).
- “Results of these studies to date give no consistent or convincing evidence of a causal relation between RF [radiofrequency] exposure and any adverse health effect” (Ahlbom et al. 2004).

There are significant differences between these statements. The last one claims that there is no “consistent or convincing evidence” (whatever this may be) of a causal relation. Hence, it points mainly to the lack of knowledge accumulated so far. The second one goes a step further: It claims that risk assessment based on the weight-of-evidence approach [as applied by the U.S. Environmental Protection Agency (U.S. EPA 1999) or the International Agency for Research on Cancer (IARC 2004)] leads to the conclusion of no causal relationship. However, there is no category of this type in the weight-of-evidence approaches. Either the category “not likely carcinogenic to humans” (U.S. EPA 1999) or “evidence suggesting lack of carcinogenicity” (IARC 2004) may be used. Because of the by far higher demands on quality and size of studies set out to dismiss the assumption of carcinogenicity, there is an inherent imbalance of classification concerning carcinogenicity and lack of carcinogenicity. The first statement goes still further: It claims that an analysis based on the Bradford Hill criteria confirms that there is no causal relationship. Because the only Bradford Hill criterion that is essential is “temporal relation,” the only way to confirm—based on these so-called criteria—that there is no causal relation is to demonstrate that exposure commenced after disease onset. All other evidence may reduce the weight in favor of a causal relationship but cannot confirm that there is no causal relationship.

## Are There Criteria for Causation?

During the past decades, Bradford Hill’s criteria have played almost the same role in occupational and environmental risk assessment as Koch’s postulates for microbiology (Koch 1882). As was the case with Koch’s postulates, which cannot be fulfilled for many infectious agents, so Bradford Hill’s criteria are supportive (for the assumption of a causal relation) only if fulfilled, but cannot be used to dismiss the assumption of a causal relation. It is a complete misinterpretation of the nine issues considered by Bradford Hill that they can be a type of checklist to establish causation. But it may turn out that they owe their popularity, still persisting after 40 years, exactly to this misconception.

Because the definition of a disease cause given above affords the existence of mutually exclusive conditions, in a strict sense, causation can be indicated only by (experimental) production and control of all (relevant) conditions. This, however, leads to ethical problems if the factor is potentially debilitating or lethal. And it is practically impossible if the latency is long, as it is for chronic diseases. Resorting to animal experimentation can reduce some of these problems but introduces new ones, because inference from results in animals to effects in humans is far from trivial. Hence, we are often left with a number of problems that cannot be optimally solved, and therefore there is no set of criteria that, if fulfilled, would result in attributing a factor as either causally related or not. This does not mean that we cannot, to the best of our present knowledge, come to a decision concerning the relationship of an agent and a disease. Or, as Bradford Hill (1965) said 40 years ago:

All scientific work is incomplete—whether it be observational or experimental. All scientific work is liable to be upset or modified by advancing knowledge. That does not confer upon us a freedom to ignore the knowledge we already have, or to postpone the action that it appears to demand at a given time.

## A Pragmatic Approach

Concerning a particular chemical or physical factor, general medical knowledge may suffice to attribute it as harmful and as causing illness or death (but even in extreme cases such derivations may not be altogether valid—e.g., the statement that it is impossible to climb Mt. Everest without respiratory aid). But in a developed society, obviously, hazardous conditions are likely to have been detected already and are subject to an individual and/or public risk–benefit evaluation. So we are dealing with either less obvious hazards or those that occur only rarely or in a small proportion of the population. The evidence may stem from all kinds of sources, but often we start only from the pessimistic assumption that an agent either not present in the natural environment or present only at much lower levels may be harmful to health. Or it may be that during routine surveillance, a high prevalence of a (rare) disease is observed that coincides with a (rare) environmental condition. How should we come to a conclusion whether the suspected environmental condition is causing disease? It might be worthwhile to stress that there are cases where we do not need the verdict of causation before we take action (e.g., a not very important food additive may be banned on weak evidence of harmful effects). An important part, and a much ignored one, of Bradford Hill’s article deals with such situations, as Phillips and Goodman (2004) pointed out.

Starting from the definition of a disease cause stated above, it is obvious that three main issues need to be addressed (to simplify the discussion, let us speak of the set of exclusive conditions as of an agent or determinant A):

- Is the probability of the disease conditional on the presence of A higher than in the absence of A? (association)
- Is the set of conditions to which the source populations are exposed sufficiently similar except for A? (environmental equivalence)
- Are the features of the populations that differ with respect to exposure to A such that, for the problem under investigation, they can be considered equivalent? (population equivalence).

### Association

Although we can to some degree rely on statistical decision theory concerning an observed difference, some problems need to be addressed: First, there are cases where we observe an incidence only in those exposed to A and contrast it to the overall incidence in the population (as was the case with hepatic angiosarcoma in workers exposed to vinyl chloride monomer). If the disease is extremely rare in the population, it may not be feasible to do a conventional epidemiologic study. However, if a plausible mechanism of action can be delineated, the observation of an unexpectedly high incidence of the disease may suffice for a verdict of causation. Second, in the case–control approach, we estimate not the conditional probabilities of the disease but their ratio. Furthermore, it is questionable whether statistical decision theory based on random sampling can be applied without further consideration. Typically, all cases of the target disease occurring within a specified region (or even only those diagnosed in one or several hospitals) and during a specified period of time are intentionally included, and only controls are sampled (either from the population or from hospital cases presenting with other than the target disease). To apply statistical decision theory, we have to assume that the cases are a random sample from the distribution of all samples related to all time/space intervals. Furthermore, the population from which the cases and controls originate has, in general, not been stable during the relevant past. Cases of the target disease that occurred before study onset are not included, and also migration in and out of the target area may play an important role, as might deaths from other and maybe related causes. Because of these circumstances and the additional problem of reliably assessing the presence of A retrospectively, case–control studies are often denied the potential to form the basis of a causal interpretation. However, this is exaggerating the difficulties associated with this study type. Especially if several case–control studies from different areas and time periods are available, a generalization about the ratio of incidences can be made if the different sources of bias have been thoroughly addressed. Finally, even if the relative risk (whether estimated from rate ratios, odds ratios, or hazard ratios) is high, statistical significance may not be reached if the number of cases exposed to A is low.

### Environmental equivalence

Ideally, those exposed to A should share the same conditions, besides A, with those not exposed to A. If not, all relevant conditions that are potentially related to both A and the outcome (i.e., confounding conditions) must be included in the data set to account for them in the analysis. Failing to do so—that is, controlling for some but not others—may increase confounding instead of removing it (e.g., Maldonado and Greenland 2001); on the other hand, controlling for a variable that is downstream of A may remove the effect of A (Kaufman and Poole 2000). Because the number of potentially confounding factors is indefinite and judgment about the degree of similarity between environmental conditions depends on limited experience, there is always the possibility that an observed association is due to confounding. On the other hand, the mere suspicion that an observed association is due to confounding does not conform to scientific reasoning because it cannot be refuted by a finite sequence of empirical tests. Analysis of uncontrolled confounding (Greenland 2003; Robins et al. 1999) can give an idea about the strength of the association between the confounding variable and both A and the outcome required to substantially alter inferences about the existence of an association between A and the outcome. These approaches may replace the earlier procedures, as already applied by Bradford Hill.

### Population equivalence

The counter-factual approach to causality (last statement in Table 1), although of questionable empirical content, has great heuristic strengths. A counterfactual cause is defined as something that leads to a difference in the disease propensity with respect to the same target (population). Although, of course, it is then impossible to ever empirically demonstrate such a cause, it points to the importance of considering all features of the populations that are substitutes for the target exposed to A or not exposed to A, respectively. Ideally, all features of these substitutes should be equal. However, this would afford restriction to homozygous twin studies with twins who shared the same experiences except for exposure to A. However, for practical purposes, it will suffice to demonstrate equivalence with respect to the features that determine susceptibility to A, disposition to develop the target disease, and the interaction between disposition and susceptibility (i.e., the joint distribution of these features).

Unfortunately, as a National Cancer Institute workshop has stressed (Carbone et al. 2004), there is insufficient evidence to stratify populations based on susceptibility to develop cancer. For other chronic diseases, such as atherosclerosis, Alzheimer’s disease, and obstructive pulmonary disease, there might be even fewer evidence-based criteria for disposition and susceptibility. Therefore, a still more modest approach must be followed that is embedded in the universal scientific scheme of bold trial-and-error correction. As a minimum requirement, we must address the features that are known to be related to disease incidence (in most cases, age will be among these features); features that indicate early steps of the target disease (e.g., polyposis for colon cancer), thereby keeping in mind that agent A may be effective only during certain steps of the pathologic process; and features that may determine the potential to counteract or aggravate the disease (e.g., social class). Scientific discussion may reveal that potentially important features have been left out. In this case, considerations of the potential bias thereby introduced may reveal that the effect of A has been underestimated (e.g., if those exposed to A can be considered less prone to develop the target disease). If the investigation resulted in a positive association between A and the target disease, we might conclude that no further investigation is needed; if, on the other hand, no association was revealed, there is indeed a need for error correction. An analogue procedure follows from a suspected overestimation of the association.

Environmental equivalence and population equivalence are usually termed the *ceteris paribus* condition and are often jointly discussed. It is, however, important to discriminate between environmental and population characteristics. Only the former can be targets of change; the latter, although not stationary at all, must be taken as side conditions that can be controlled only by active selection. It is also important to consider self-selection processes in observational studies where features of the environment may determine to some degree features of the population and vice versa.

It goes without saying that all investigations that are assessed for a causal interpretation must be scrutinized for potential biases (especially exposure and outcome misclassification and response or observer bias). However, it is insufficient merely to point to a potential bias without considering the effect this bias may have had on the results. For example, in cohort studies, exposure misclassification can lead to a bias only in the opposite direction of the reported association.

Under the precondition that all investigations have been thoroughly assessed concerning association, environmental equivalence, and population equivalence, and potential biases, and still the following set of statements can be derived, then it is reasonable to allocate A among the potentially causal factors of the target disease:

- The temporal relationship between exposure to A and disease onset (or diagnosis) conforms to what is known about the natural history of the disease.
- There is an association between exposure to A and the target disease.
- Environmental characteristics in which exposed and unexposed populations live can be considered equivalent during the etiologically relevant period except for A.
- Characteristics of exposed and unexposed populations are sufficiently similar to consider them equivalent.

Only the first two statements are essential; the latter two can be substituted by evidence from experimental or other research demonstrating a mechanism of action that does not depend on individual characteristics or environmental factors. Furthermore, if it is impossible to demonstrate the equivalence condition, then other considerations and evidence can be substituted to support the assumption of a causal relation (see below).

Temporal relation, association, and environmental and population equivalence suffice for a verdict of potential causation. This assertion can only be refuted by the following:

- Evidence that demonstrates that A is a downstream condition of some other factor B (e.g., *Helicobacter pylori* infection instead of gastritis as a potential causal factor for atherosclerosis)
- Evidence that A is associated with B, the essential causal agent (e.g., technical tetrachloroethene contaminated with epoxybutane)
- Evidence that essential side conditions have been overlooked that need to be present to make A effective or to make non-A preventive (e.g., a specific receptor phenotype).

It is not necessary to demonstrate a mechanism of action. Bradford Hill (1965) and others pointed to the landmark 1854 study of John Snow, who demonstrated that the rate of cholera deaths in London was 14 times higher in households supplied with water from the Southwark and Vauxhall Company compared with households supplied with water from the Lambeth Company (Snow 1855). Although Snow suspected a living organism contaminating drinking water by proximity to sewage, another 30 years elapsed before Robert Koch isolated *Vibrio cholerae*, and more than 100 years before the mechanism of action of the cholera toxin was established. The original observation of Snow sufficed to state that something in the water supplied by one company potentially caused cholera and to take appropriate action (closing the pump), and there was no need to wait until a mechanism of action had been demonstrated (thereby probably sacrificing the lives of thousands of people). However, if a mechanism of action can be established, the requirements for epidemiologic evidence outlined above can be somewhat relaxed.

Because of difficulties inherent in observational studies, it may be impossible to demonstrate environmental and/or population equivalence to a sufficient degree, and therefore additional evidence and considerations are necessary to support the notion of a causal relation between agent A and the target disease. There is no possible evidence beyond the three points stated above that will refute epidemiologic evidence in favor of a causal relation besides more and “better” epidemiologic evidence. Stakeholders tend to “flood” the scientific literature with inconclusive (powerless and/or biased) studies in the hope that the balance of evidence will turn in favor of a less strong association between agent A and the target disease. Assessment of evidence must take this into consideration and make proper use of such information (which in most cases will result in disregarding it altogether).

There is an extensive literature about “criteria” for causal inferences in the health sciences, most of which goes back to the seminal work of Bradford Hill (1965) and Mervyn Susser (1973). Although neither author meant to establish a checklist, but only to formulate issues that aid in this task, application has been more or less schematically following these criteria. However, there is no rule that can guide the decision. How many of the criteria must be fulfilled? Is one counting more than the other? What to do if none is fulfilled? There is no straightforward answer to these questions, and every single case merits its own specific line of argumentation.

Tables 2 and 3 propose a dialogue approach to causal inference. It is assumed that epidemiologic evidence has been put forward that is evaluated along the criteria outlined above. A scientific dialogue of conjecture and refutation at first tries to dismiss the notion of a causal relation between agent/determinant A and disease D along the four issues “temporal relation,” “association,” “environmental equivalence,” and “population equivalence.” There are valid and invalid counterarguments. If the dialogue ends without valid counterarguments, no further evidence for the verdict of causation is necessary. More often than not, epidemiologic evidence will be insufficient (e.g., due to short duration of exposure). In this case, other evidence may support or weaken the assumption of a causal relation between A and D. The most important of these arguments favoring or against causation are shown in Table 3. Arguments against causation are often not symmetrical to arguments in favor of causation. For example, a long-term experiment in animals that results in a higher incidence of the target disease in exposed animals supports causal inference, whereas a negative result does not support the assumption of no causal relation, because the tested species or strain may lack a decisive feature (e.g., an enzyme) that is present in humans and necessary for A to produce D. There are, however, cases where a positive result in animal experiments cannot be taken as evidence for causation because of processes not present in humans.

Most risk assessment procedures demand that for chronic diseases such as cancer there must be epidemiologic evidence before an extrinsic agent can be ascribed a hazardous potential for human health. Considering the long latencies involved in these diseases, there is a need to define procedures that give answers about a potential causal relationship in a more rapid fashion. Traditional epidemiologic evidence can be provided only *ex post*, when the health impairment has already occurred in a significant fraction of the exposed population. There is an urgent need to connect the disciplines of molecular biology and epidemiology (Carbone et al. 2004). Such collaboration should result in *a*) a better characterization of the study participants with respect to susceptibility and *b*) early markers of responses to the agent in question that can be assessed long before occurrence of manifest disease. With regard to such new approaches, it is of paramount importance to investigate the mechanism of interaction of the extrinsic agent with the organism in order to define potential cofactors and sensitive end points. For chemical substances, *in silico* methods and structure–activity considerations may provide first answers to a potential path of action (e.g., binding to a receptor). For physical factors such as electromagnetic fields, knowledge is more limited, and new approaches must be designed.

Despite its metaphysical character, the principle of causation or, more specifically, the notion that every disease has a cause has been of great heuristic value and likely will govern our future endeavors for better understanding of the relationship between the environment and human health until we have accumulated more knowledge and may describe the process by a system of equations. However, the complexity of the problem may be too great ever to lend itself to complete description.

## Figures and Tables

**Table 1:** Definitions of causation from the epidemiologic literature (modified from Parascandola and Weed 2001).

Definition	Main criticism
A cause is something that produces or creates an effect.	Tautological because “production” and “creation” are synonyms of “causation”
A cause is a condition without which the effect cannot occur.	Only very few diseases could then have a cause^a^
A cause is a condition with which the effect must occur.	Again, only few diseases could then have a cause^b^
A cause is made up of several components, no single one of which is sufficient of its own, which taken together must lead to the effect.	Introduces unnecessary complexity in cases of simple dose response and in cases of interaction between components
A cause is a condition that increases the probability of occurrence of the effect.	Does not distinguish between an association and a “cause”^c^
A cause is a condition that, if present, makes a difference in (the probability of) the outcome.	Is, in the strict sense, unprovable because there is only one world and one cannot observe it twice—once with and once without the condition

a Many disease definitions already include a cause (e.g., AIDS is a clinical syndrome in the presence of HIV infection of CD4 cells), but this must not be confused with a necessary cause. All clinical symptoms that occur in AIDS patients can have a variety of other “causes.”

b For example, falling from the 27th floor onto the pavement is not a necessary cause for breaking the skull because many other processes can lead to this effect; however, it can be seen as a sufficient cause. Except for injuries due to extreme physical or chemical conditions and exposure to extremely contagious infectious agents that lead to death (e.g., rabies) or do not result in immunity (e.g., gonorrhea), there are no sufficient causes in this strict sense.

c Following this definition, male sex would be a cause of lung cancer.

**Table 2:** A pragmatic dialogue approach to causal inferences about an agent or determinant A with respect to a disease D: Evidence from epidemiologic studies.

	Counterarguments	
In favor of causation	Valid	Invalid
Temporal relation	Exposure to A commenced after onset of D.	No mechanism of action of A on any or all stages of D has been established.
Association	A is a downstream factor of agent/determinant B that has been indicated as a causal factor of D.	Exposure to A has not been precisely assessed.
	A is associated with B that has been indicated as a causal factor of D.	There could be exposure misclassification.
	There is differential bias (response or observer bias) in the direction of an association between A and D.	There is a potential bias (response or observer bias) with unknown effect on the association between A and D.
	There has been differential disease misclassification in cohort studies.	There has been disease misclassification in case–control studies but not associated with exposure.
Environmental equivalence	Confounding conditions with a combined effect exceeding that of agent A have not been considered.	There could have been confounding.
Population equivalence	A is associated with selection into the study population. Risk of A applies only to a subgroup of the population. Exposure is associated with *a priori* risk to develop the disease.	There is a potential selection bias with unknown effect on the association between A and D.

At this stage no further evidence is necessary for establishing causation unless valid counterarguments have been put forward.

**Table 3:** A pragmatic dialogue approach to causal inferences about an agent or determinant A with respect to a disease D: Evidence increasing or decreasing confidence in a potential causal relation between A and D.

Type of evidence	Increasing confidence	Decreasing confidence
From prior knowledge	Results conform to predictions from theoretical considerations and/or prior knowledge about specificity of outcome, specificity of type of exposure, or specificity regarding the outcome in different subgroups of the population.	Although there are sound arguments for specificity of outcome, specificity of type of exposure, or specificity regarding the outcome in different subgroups of the population, data do not conform to these expectations.
	Association between A and D is coherent with biologic knowledge and/or a plausible mechanistic model of action can be delineated.	There is knowledge about mechanism of action that indicates lack of effect of A on D.
From epidemiology	Strength of association between A and D exceeds that of potential confounders.	There are known confounders not considered in existing investigations strong enough to explain the observed effect.
	Association between A and D is consistently observed in different populations, with different types of studies, or in different time intervals.	There is substantial heterogeneity in the effect of A on D in different populations, different study types, or different time intervals.
	Manipulating A in the population changes pattern and/or frequency of D.	Manipulating A in the population does not affect occurrence of D.
	In the case a meaningful meter of the “dose” of A can be defined, there exists a dose–response relationship.	A meaningful “dose” meter can be defined but the relationship between “dose” and response is not monotonous.
From animal studies	Long-term animal studies in different species indicate an association between A and D (or D′, an analogue of D in these species).	There exist animal models of the disease D, and in none of these models A is effective.
	A enhances the effect of a known pathogen B.	No promoting or antagonizing effect of A with a variety of other agents could be found in different exposure regimes relevant for human exposures.
	In animal experiments, intermediate steps of the pathogenic process can be evoked by exposure to A.	In different species that are sensitive to other exposures producing effects expected to be similar to those of A, the latter is ineffective.
From *in vitro* studies	Exposed cells or tissues react or get damaged by exposure to A consistent with the pathogenic process of D.	In cell lines or tissues sensitive to exposures similar to A, no effect of exposure to A is found.
	Upstream events can be observed by exposure to A that may lead to D in the intact organism.	No changes in cellular processes or alterations of signaling pathways can be evoked by exposure to A.
	A enhances the effect of a known cellular pathogen B.	No promoting or antagonizing effect of A with a variety of other agents could be found.
